# Mechanics and regulation of cytokinetic abscission

**DOI:** 10.3389/fcell.2022.1046617

**Published:** 2022-11-24

**Authors:** Virginia Andrade, Arnaud Echard

**Affiliations:** ^1^ CNRS UMR3691, Membrane Traffic and Cell Division Unit, Institut Pasteur, Université Paris Cité, Paris, France; ^2^ Collège Doctoral, Sorbonne Université, Paris, France

**Keywords:** cytokinesis, abscission, actin, myosin II, ESCRT, cell mechanics, caveolae, cell division

## Abstract

Cytokinetic abscission leads to the physical cut of the intercellular bridge (ICB) connecting the daughter cells and concludes cell division. In different animal cells, it is well established that the ESCRT-III machinery is responsible for the constriction and scission of the ICB. Here, we review the mechanical context of abscission. We first summarize the evidence that the ICB is initially under high tension and explain why, paradoxically, this can inhibit abscission in epithelial cells by impacting on ESCRT-III assembly. We next detail the different mechanisms that have been recently identified to release ICB tension and trigger abscission. Finally, we discuss whether traction-induced mechanical cell rupture could represent an ancient alternative mechanism of abscission and suggest future research avenues to further understand the role of mechanics in regulating abscission.

## 1 Introduction

**FIGURE 1:**
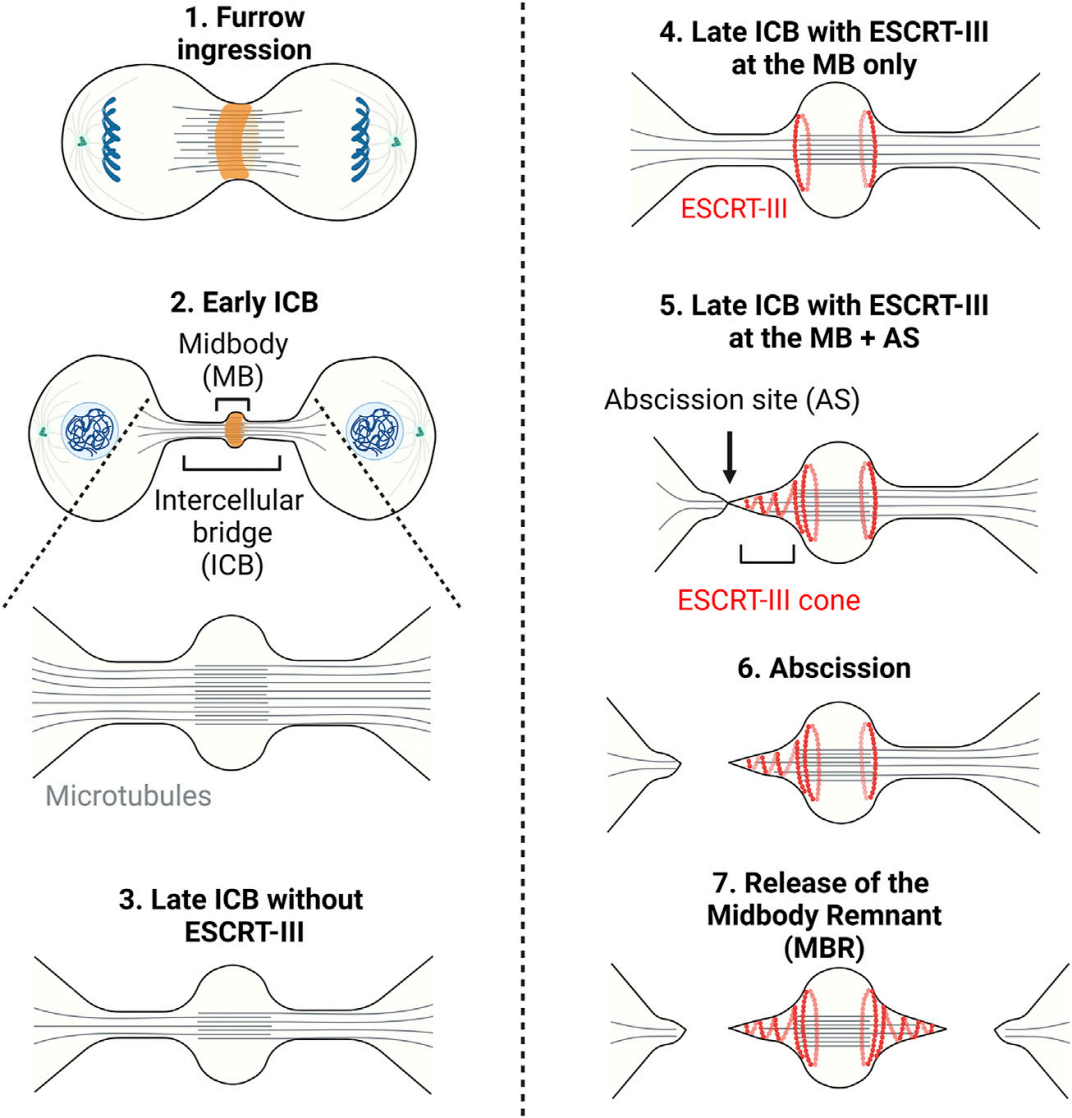
– Cytokinesis: A multi-step process. Step1 - Contraction of an actomyosin ring (orange) during furrow ingression. Step2 - Formation of the intercellular bridge (ICB) and midbody containing antiparallel microtubules (grey lines). Step3 - Thinning of the ICB. Step4 - Recruitment of the ESCRT-III to the midbody (MB). Step5 - Polymerization of ESCRT-III (red curves) on one side of the midbody and severing of the microtubules. Step6/7—Scission of the ICB membrane (abscission) and release of the midbody remnant (Step7). Adapted from (Andrade et al., 2022).

Cytokinesis is the process by which a mother cell is physically separated into two daughter cells after mitosis. In animal cells, it starts in anaphase with the contraction of a membrane-associated actomyosin ring at the midplane of the cell leading to the ingression of a cleavage furrow (Green et al., 2012; D'Avino et al., 2015; Srivastava et al., 2016) (Figure 1, step 1). Ingression of the furrow is followed by the formation of an intercellular bridge (ICB) connecting the daughter cells, at the center of which is found a prominent, protein rich structure, the midbody (Figure 1, steps 2–5). Abscission, the physical cut of the ICB, eventually occurs and concludes cytokinesis (Figure 1, step 6). Abscission requires the fission of the plasma membrane of the ICB and is preceded by the local clearance of the microtubules (MTs) filling the ICB and of the actin filaments (F-actin) present in the ICB (Figure 1, step 5) (Mierzwa and Gerlich, 2014; Addi et al., 2018).

It was long thought that after furrow ingression the ICB is mechanically ruptured. This was suggested by the observations that the ICB gets thinner as cytokinesis progresses and that the dividing cells exert traction forces on the substrate (Mullins and Biesele, 1977; Burton and Taylor, 1997). These forces were proposed to be important for each daughter cell to pull on the ICB and, consequently, to drive abscission. However, in 2007, the finding that the membrane remodeling machinery ESCRT (Endosomal Complex Required for Transport) is recruited at the ICB and is required for abscission questioned the concept of mechanically-driven cell separation (Carlton and Martin-Serrano, 2007; Morita et al., 2007). Abscission was then proposed to be topologically equivalent to other membrane fission events executed by the ESCRT machinery, such as intraluminal vesicle (ILV) formation in late endosomes/multivesicular bodies (MVBs), and Human Immunodeficiency Virus (HIV) budding at the plasma membrane (Christ et al., 2017; Scourfield and Martin-Serrano, 2017; Stoten and Carlton, 2017). Notably, members of the ESCRT-III family can polymerize into filaments and are likely responsible for the constriction and scission of the membrane. As the ICB matures, ESCRT-III components assemble at the midbody and then constrict the plasma membrane on one side of the midbody (Mierzwa and Gerlich, 2014; Addi et al., 2018) (Figure 1, step 5). This eventually leads to the fission at the abscission site (Figure 1, step 6). The same process usually occurs on the other side of the midbody, ending up in the formation of a midbody remnant (Figure 1, step 7) (Crowell et al., 2013; Crowell et al., 2014; Peterman and Prekeris, 2019). These findings underline that the physical cut of the ICB depends on a dedicated molecular machinery, which turns out to be highly regulated.

Paradoxically, using HeLa cells, it was later reported that the pulling forces exerted by the daughter cells on the ICB—which induce high tension at the ICB—inhibit abscission instead of promoting it (Lafaurie-Janvore et al., 2013). Thus, tension release was proposed to trigger a sequence of events leading to cytokinetic abscission. As originally defined in (Lafaurie-Janvore et al., 2013), the term ICB tension is used here and thereafter in a broad sense, to describe the fact the ICB experiences pulling forces. How ICB tension is related to membrane tension and to cortical tension will be detailed later. In this Review, we will focus on the significant progress made recently to understand the mechanical regulation of abscission. We will first summarize the accumulating evidence that ICB tension inhibits abscission, at least in some epithelial cells. We will next explain why a drop in tension promotes abscission, by favoring ESCRT-III assembly at the abscission site. We will also describe the first molecular and cellular complexes identified so far that control ICB tension in late cytokinetic steps. We will argue that the sequential increase and decrease of ICB tension are actually required in these cells for ICB maturation and abscission, respectively. We will finally discuss the possible physiological relevance of a tension-dependent control of abscission, and raise key unanswered questions in this relatively new field.

## 2 The ICB initially experiences pulling forces but abscission requires a drop in the ICB tension

### 2.1 The ICB is under tension

The molecular machinery that leads to abscission, the ESCRT machinery, has been well studied. However, the mechanical context of this process and how the recruitment of the abscission machinery depends on tension remained poorly studied until recently. Using Swiss 3T3 fibroblasts, it was originally shown that the daughter cells exert traction forces on a compliant silicone deformable substrate while they migrate apart and respread on the substrate after furrow ingression (Burton and Taylor, 1997). In most cells analyzed, forces exerted by the daughter cells to the substrate rise throughout cell separation and were thus proposed to contribute to the thinning and eventual breakage of the ICB (Burton and Taylor, 1997). While this study demonstrated that fibroblasts, during the migration phase, exert pulling forces on the substrate in the order of hundreds of nanonewtons (nN), the forces applied on the ICB could not be measured. Confirming this study, human epithelial HeLa cells pull on the substrate after furrow ingression, and the contractile energy of daughter cell doublets—in the order of 0.5–1 x 10^−14^ J—can be measured by traction force microscopy (TFM) (Lafaurie-Janvore et al., 2013). In addition, tension at the ICB was investigated using TFM combined with laser ablation (Lafaurie-Janvore et al., 2013). Measuring the displacement of fluorescent microbeads embedded in polyacrylamide gels upon laser-mediated severing of the ICB allowed to infer the magnitude of forces exerted on the bridge. Using this approach, the authors showed that ICBs experience pulling forces in the nN range (1.4 ± 0.2 nN) (Lafaurie-Janvore et al., 2013). Similar values were obtained by measuring the speed of retraction/recoil of the ICB immediately after laser ablation, assuming that the ICB behaves like a rigid body and that the retraction of the ICB reflects the viscoelastic relaxation of the cell at short time scales (Lafaurie-Janvore et al., 2013).

**FIGURE 2:**
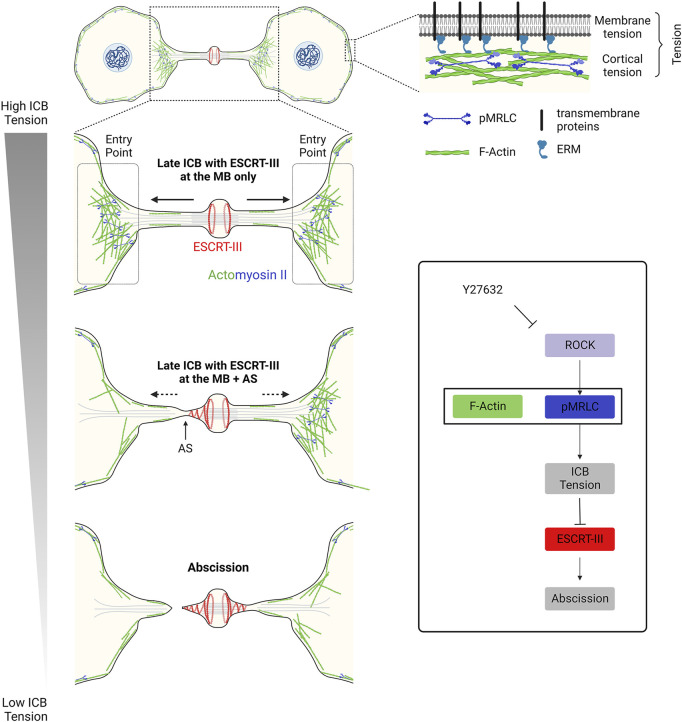
– Decrease in tension at the ICB triggers ESCRT-III polymerization and successful abscission in epithelial cells. Left panel: Decrease in the ICB tension and reduction of actomyosin contractility at the Entry Points (EPs) allow ESCRT-III polymerization, as cells progress towards abscission. Top right panel: Tension results from a combination of membrane tension and actomyosin-dependent cortical tension. Box: Schematic summarizing the contribution of the actomyosin network to the ICB tension, ESCRT-III polymerization and abscission. Y27632: drug that inhibits ROCK and prevents the phosphorylation (activation) of myosin II.

A key question is to understand the origin of the forces applied to the ICB putting it under tension after furrow ingression. Since ICB tension correlates with daughter cell separation speed, it was proposed that actomyosin-dependent cellular contractility and motility observed when cells respread on the substrate contribute to the generation of pulling forces at the ICB (Lafaurie-Janvore et al., 2013). Accordingly, inhibiting the overall cellular actomyosin contractility using the Rho-kinase (ROCK) inhibitor Y27632 at high doses abolishes ICB tension (Lafaurie-Janvore et al., 2013) (Figure 2, bottom right panel). More locally at the ICB, one can envisage several sources of tension (Figure 2, upper right panel). A first source of tension to consider is membrane tension. Although the nomenclature can vary among authors (see (Sens and Plastino, 2015; Sitarska and Diz-Muñoz, 2020)), membrane tension can be defined as the combination of the in-plane plasma membrane tension and the tight connection of the plasma membrane to the underlying actin cortex. This occurs notably *via* anillin, transmembrane proteins (e.g. CLIC1/4) and ERM (Ezrin-Radixin-Moesin) in late ICBs (Carreno et al., 2008; Kunda et al., 2008; Fehon et al., 2010; Carim et al., 2020; Uretmen Kagiali et al., 2020). In-plane plasma membrane tension depends on the deformability of the lipid bilayer and can be controlled by many factors such as lipid composition, osmotic pressure, the presence of membrane invaginations (such as caveolae, see Section 3.2), spreading and adhesion to the substrate of the daughter cells and endo/exocytosis events at the plasma membrane (Sens and Plastino, 2015). Whether changes in the in-plane membrane tension spread rapidly across the whole plasma membrane or can be locally restricted is debated (Shi et al., 2018; DeBelly et al., 2020). This opens the possibility that the membrane tension at the ICB might be different from the rest of the cell and locally regulated, although measurements at different cell locations—at the ICB vs. cell body—lead to similar results (Lafaurie-Janvore et al., 2013). In any case, using membrane tube pulling experiments, it was shown that membrane tension can account for a third to half of the forces (0.4–0.8 pN) measured at the ICB (1.4 nN) (Lafaurie-Janvore et al., 2013). A second source of tension to consider is the cortical tension, since the actin cortex itself is also under tension (Sens and Plastino, 2015; Sitarska and Diz-Muñoz, 2020). The cortical tension can be locally regulated and largely depends on the thickness of the actin cortex and the level of penetration of activated myosin II (Truong Quang et al., 2021). Interestingly, a pool of cortical F-actin and myosin II was recently characterized at the interface between the ICB and the cell bodies (at regions named “Entry points” or “EPs”, Figure 2, left panels) and could directly contribute to the observed tension at the ICB (Andrade et al., 2022). However, the relative contribution of the different cellular pools of actomyosin (at EPs vs. in the cell bodies or potentially within the ICB, see below) is unknown and remains to be clarified. A third potential source of tension to consider is the tension of other cytoskeletal elements of the ICB, notably the numerous MTs that are present all along the ICB and tightly connected to the daughter cell bodies. Upon severing of the ICB MTs before abscission, ICB MT bundles are seen to retract into the cell bodies, suggesting that they are also under tension (Elia et al., 2011; Lafaurie-Janvore et al., 2013; Addi et al., 2020). In summary, membrane tension appears to have an important contribution in ICB tension but other sources must have a role.

### 2.2 Tension release at the ICB triggers abscission in epithelial cells

Several lines of evidence indicate that abscission is inhibited when ICBs experience high tension in HeLa cells (Lafaurie-Janvore et al., 2013). First, using fibronectin-coated micropatterns to confine cell migration, it was shown that the more daughter cells can move apart, the more abscission is delayed. It should be highlighted that in this study and in many other studies in the field, the timing of abscission is defined by the cut of the MTs on one side of the midbody and not by the actual membrane scission. These two events might not be simultaneous. Second, cells with straight ICBs (based on α-tubulin-GFP fluorescence), which likely represent tensed bridges, show delayed abscission. On the contrary, cells cultured at high density display short and compressed, buckled ICBs, which correlate with rapid abscission times. Third, there is a strong correlation between fast speed of daughter cell migration and spreading, high ICB tension and long abscission time. Fourth, TFM measurements showed that daughter cells with high contractility pull more on the substrate and display delayed abscission. All together, these results suggest that a drop in the ICB tension could be required to trigger abscission (Lafaurie-Janvore et al., 2013).

Such a release of ICB tension can be actually experimentally measured in wild type dividing cells and shortly precedes abscission (Lafaurie-Janvore et al., 2013). Indeed, based on laser ablation experiments, it was observed that ICBs on the brink of abscission—inferred from small bridge diameter and α-tubulin-GFP fluorescence—are under lower tension than average ICBs. In addition, using time-lapse microscopy, the authors noticed that daughter cells stop moving apart 10 min before abscission, before migrating toward each other, as demonstrated by compressed ICB labelled with α-tubulin-GFP. Abscission reproducibly occurs 10–15 min after this tension release.

To directly demonstrate that a drop of the ICB tension can trigger abscission, two types of experiments were performed (Lafaurie-Janvore et al., 2013). First, Y27632 was used to abolish the ICB tension. This treatment induces abscission within 20 min, compared to 65 min in control situation. Second, laser ablation was performed on one side of the midbody to rapidly decrease the ICB tension. This leads to abscission on the other side of the midbody shortly (approximately 25 min) after, which is not observed by ablating cells outside the ICB. This is the most direct proof so far that pulling forces in the ICB inhibit abscission. All together, these results strongly suggest that 1) a drop in ICB tension is required to trigger abscission and 2) ICB tension must be tightly regulated by specific cellular complexes, as we shall see in Section 3.

## 3 ICB tension controls the polymerization of the ESCRT-III at the abscission site

### 3.1 Sequential recruitment of ESCRT-III to the ICB

**FIGURE 3:**
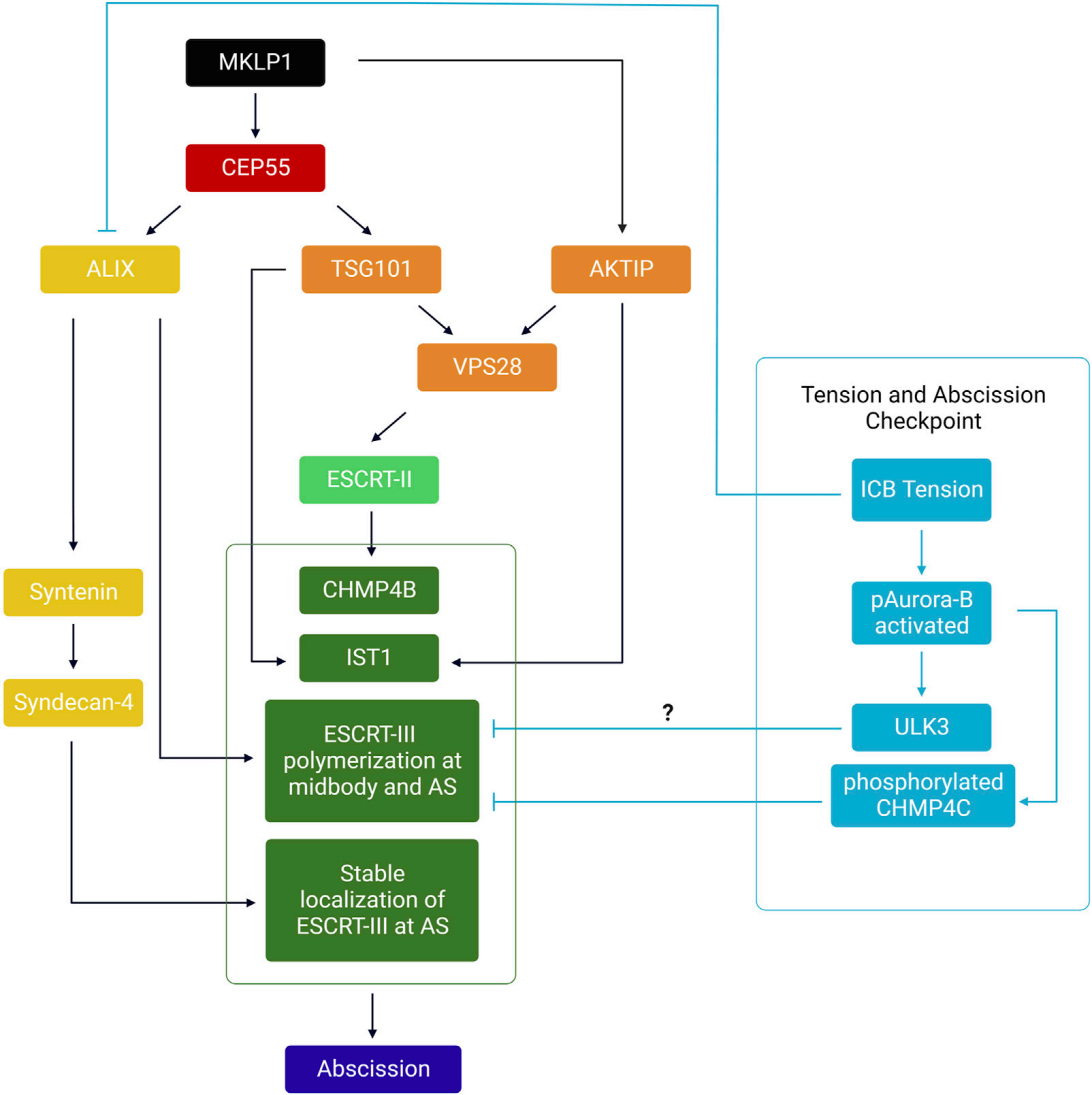
– Summary of the hierarchical events involved in the recruitment of the ESCRT-III machinery at the midbody and abscission site. MKLP1 at the midbody recruits both CEP55 and AKTIP. CEP55 directly recruits ALIX and TSG101, which act as two parallel pathways to recruit ESCRT-I/-II/-III at the midbody. ESCRT-III polymerizes to the abscission site (AS). Syntenin and Syndecan-4 stabilize ESCRT-III filaments at the AS. Orange: ESCRT-I related proteins; Light green: ESCRT-II components; Dark green: ESCRT-III components. Yellow: ALIX and associated complex (Syntenin, Syndecan-4). In cyan: Relationship between tension, the abscission checkpoint and ESCRT polymerization at the ICB. Only components of the abscission checkpoint likely involved in response to ICB tension have been depicted.

The evolutionarily conserved ESCRT machinery is composed of proteins that fall into four multimeric protein complexes: the ESCRT-0 (not involved in abscission and thus not discussed here), ESCRT-I, ESCRT-II and ESCRT-III. Proteins of the ESCRT-III family can polymerize and drive membrane fission of membrane necks from their luminal (cytosolic) side, as shown *in vivo* and *in vitro* (see these excellent reviews for details (Christ et al., 2017; Schoneberg et al., 2017; Scourfield and Martin-Serrano, 2017; Stoten and Carlton, 2017; McCullough et al., 2018; Remec Pavlin and Hurley, 2020; Vietri et al., 2020; Pfitzner et al., 2021)). During cytokinesis, most proteins of the ESCRT-III complex (CHMP1A/B, CHMP2A/B, CHMP3, CHMP4A/B/C, CHMP5, CHMP6, IST1 but not CHMP7) are first recruited at the midbody (Figure 1, step 4) and then polymerize in a cone-like structure pointing toward the abscission site located approximately 1 μm on the side of the midbody (Figure 1, step 5) (Carlton and Martin-Serrano, 2007; Morita et al., 2007; Elia et al., 2011; Guizetti et al., 2011; Capalbo et al., 2012; Carlton et al., 2012; Elia et al., 2012; Hadders et al., 2012; Hu et al., 2012; Lee et al., 2012; Schiel et al., 2012; Lafaurie-Janvore et al., 2013; Crowell et al., 2014; Goliand et al., 2014; Renshaw et al., 2014; Capalbo et al., 2016; Christ et al., 2016; Sherman et al., 2016; Fremont et al., 2017a; Mierzwa et al., 2017; Goliand et al., 2018; Addi et al., 2020; Andrade et al., 2022). In HeLa or MDCK cells, where abscission has been most studied, ESCRT-III recruitment at the midbody depends on the kinesin MKLP1 and its associated protein CEP55, which directly interacts with ESCRT-I TSG101, ESCRT-I-like AKTIP and ESCRT-III-associated ALIX (Zhao et al., 2006; Carlton and Martin-Serrano, 2007; Morita et al., 2007; Lee et al., 2008; Goliand et al., 2014; Christ et al., 2016; Merigliano et al., 2021). As depicted in the left panel of Figure 3 **,** these proteins, directly or indirectly, mediate the recruitment of ESCRT-III at the midbody, through partially redundant pathways. Furthermore, direct interactions between Septin9 (SEPT9) and TSG101 is required for the normal organization of downstream ESCRT-III components at the midbody and at the abscission site (Estey et al., 2010; Karasmanis et al., 2019). It was also recently found that ALIX, Syntenin and the transmembrane proteoglycan Syndecan-4 form a tripartite complex that stabilizes the ESCRT-III cone at the abscission site and thereby promote abscission (Addi et al., 2020) (Figure 3). Importantly, abscission requires a dynamic turnover of the ESCRT-III polymers, which is mediated by the ATPase VPS4A/B (Mierzwa et al., 2017). At the site of abscission, MTs are locally severed by the ATPase spastin—which is recruited by direct interaction with CHMP1B and IST1 (Yang et al., 2008; Agromayor et al., 2009; Connell et al., 2009; Guizetti et al., 2011; Goliand et al., 2018)— and/or MT buckling (Schiel et al., 2011). Finally, as described later, other pathways are required for clearing F-actin at the ICB, in particular at the abscission site.

### 3.2 ICB tension inhibits ESCRT-III localization at the abscission site

As mentioned in Section 2.2, in HeLa cells, reducing the ICB tension by cutting it with a laser on one side of the midbody triggers abscission on the other side of the midbody, approximately 25 min afterwards (Lafaurie-Janvore et al., 2013). Given the role of ESCRT-III in recruiting spastin and the abscission machinery, the authors used time-lapse microscopy to analyze the recruitment of the ESCRT-III component CHMP4B-GFP upon laser ablation. When the cut was performed once the ESCRT-III has already been recruited at the midbody but not yet at the abscission site, an ESCRT-III cone forms rapidly, 10 min after ablation, on the side of the midbody opposite to the cut side. The depletion of CHMP2A—a component of the ESCRT-III polymers essential for abscission—prevents the formation of this ESCRT-III cone after ablation. In addition, the MT cut induced by this ESCRT-III cone depends on the presence of spastin. This argues that the ESCRT-III cone experimentally induced by tension release behaves as the ESCRT-III cone observed at the abscission site in untreated cells. Thus, high tension in the ICB inhibits abscission by preventing the polymerization of ESCRT-III as a cone toward the abscission site. Consistent with this model, when cells that have not been experimentally manipulated start to migrate toward each other and push on their ICB—which again likely releases part of the ICB tension, as testified by bent ICB MTs—CHMP4B polymerization on the side of the midbody is also observed within 10 min. Interestingly, ablation of the ICB before the recruitment of the ESCRT-III at the midbody only leads to a transient recruitment of ESCRT-III at the midbody, without subsequent cone formation (Lafaurie-Janvore et al., 2013). This suggests that tension release is necessary but not sufficient to trigger ESCRT-III polymerization at the abscission site. It is thus likely that other proteins, not regulated by tension but required for polymerizing and stabilizing ESCRT-III must first accumulate at the midbody.

Taken together, these experiments suggest that reducing ICB tension is one of the most upstream events that triggers ESCRT-III localization at the abscission site during normal division. As described in more detail in Section 3.2, this model was recently supported by the fact that 1) mutant situations that increase ICB tension delay the accumulation of ESCRT-III at the abscission site, and 2) experimentally decreasing ICB tension in these mutant cells is sufficient to restore ESCRT-III localization at the abscission site (Andrade et al., 2022).

### 3.3 Low membrane tension favors ESCRT-III polymerization *in vitro*

How the forces acting on the ICB control ESCRT-III assembly at the abscission site is not entirely understood. As initially proposed, one possibility is that high membrane tension resulting from high pulling forces on the ICB could inhibit ESCRT-III assembly (Lafaurie-Janvore et al., 2013).

The role of membrane tension in ESCRT-III polymerization was recently addressed *in vitro*, using minimal reconstitution systems composed of purified ESCRT components (either from yeast or from mammals) and unilamellar vesicles of defined lipid composition. First evidence of an inhibitory role of membrane tension on ESCRT-dependent intraluminal vesicle formation came from a bulk-phase internalization assay (Wollert et al., 2009) using Giant Unilamellar Vesicles (GUVs) incubated with yeast ESCRT-II complex (Vps22, Vps36, Vps25), the core ESCRT-III components Vps20, Vps24, Vps2 and Snf7 (Booth et al., 2019) and Vps4 in the presence of ATP. Decreasing membrane tension using an hyperosmotic buffer (+10 mOsM) increases by 3–4 times the number of ILVs formed in this assay, both in the presence or in the absence of Vps4 (Booth et al., 2019). In a separate study, the association rate of human CHMP4B to GUVs is found to increase by 3-folds in a hypertonic buffer (500 mOsm) used to lower membrane tension (Mercier et al., 2020). In addition, when the membrane tension of GUVs hold by a micropipette is decreased by reducing aspiration (in isotonic conditions) a 2-fold increase in the binding rate of CHMP4B is observed. Moreover, the authors pulled membrane tubes from GUVs with optical tweezers to quantitatively measure membrane tension and found that the polymerization rate of CHMP4B is inversely proportional to membrane tension and is dramatically reduced above a tension threshold of ∼0.1 mN m^−1^. As the authors mentioned, this value is of interest since it is consistent with the polymerization force of Snf7 (the yeast homologue of CHMP4) measured previously (Chiaruttini et al., 2015), suggesting that membrane tension could directly compete with CHMP4B polymerization. Finally, reducing membrane tension of large unilamellar vesicles (LUVs) induces the apparition of both CHMP4B filamentous spirals and membrane tubulation *in vitro* (Mercier et al., 2020). Thus, it was proposed that a decrease in membrane tension facilitates ESCRT-III assembly and polymerization on the membrane surface, thereby promoting membrane constriction by ESCRT-III. This could be relevant *in vivo*, since hyperosmotic shock induces a rapid and transient recruitment of ALIX, CHMP1B, CHMP4B and VPS4 (but not TSG101 or CHMP3) on the membrane of low-tension endosomes (Mercier et al., 2020).

## 4 Regulators of the ICB tension and of the mechanical properties of the ICB membrane during cytokinesis

### 4.1 Actin, profilin, ROCK, myosin II and dynamic regulation of ICB tension

#### 4.1.1 Actomyosin-dependent traction forces and maturation of the ICB

As described in Section 1, daughter cells exert traction forces while respreading on the substrate after furrow ingression. The resulting tension exerted on the ICB was proposed to contribute to its reduction in diameter from 1.5 to 2 μm initially to 100–300 nm when the ESCRT machinery assembles (Mullins and Biesele, 1973; Mullins and Biesele, 1977; Burton and Taylor, 1997; Elia et al., 2011; Lafaurie-Janvore et al., 2013). Several lines of evidence suggest that actomyosin-dependent contractility is critical for this maturation step of the ICB.

First, the actin binding protein Profilin—which promotes actin polymerization—is required both to develop strong traction forces on the substrate after furrow ingression and for abscission in mouse chondrocytes (Bottcher et al., 2009). Deletion of the gene encoding Profilin 1 in chondrocytes does not impair furrow ingression but leads to post-furrowing ICB instability and abscission defects, resulting in binucleation both in culture cells and *in vivo*. TFM experiments showed that Profilin-deficient cells exert reduced and non-directional traction forces while cells migrate apart and respread, compared to control cells. This was associated with decreased stress fiber assembly and focal adhesion formation/maturation resulting from impaired formin-mediated actin filament elongation (Bottcher et al., 2009). In Profilin-deficient cells, it would be interesting to directly measure whether the pulling forces exerted on the ICB are decreased and whether the reduction in the ICB diameter is insufficient to allow ESCRT-III polymerization (see below).

Second, the inhibition of myosin II activity in HeLa cells by the drug blebbistatin at doses that do not impair furrow ingression delays abscission. This is associated with an inhibition of the thinning of the ICB, measured by tubulin staining, and results in ICBs with diameters >1 μm (Wang et al., 2019). Thus, actin polymerization and myosin II activity both participate to the maturation of the ICB and reduction of its diameter.

#### 4.1.2 Actomyosin II-dependent constriction defines the future abscission site and favors F-actin clearance from ICBs

It was shown that endogenous myosin IIB concentrates in early ICBs after furrow ingression as two rings, on both sides of the midbody in HeLa cells (Wang et al., 2019). These sites of accumulation correspond to early constriction zones of the ICB that have been named in this study “sites of constrictions” (SOCs), and are proposed to become the future “sites of abscission” (SOAs) (Wang et al., 2019). Functionally, the motor activity of myosin II is required to locally pinch the ICB at SOCs, contributing to the local thinning of the ICB. In presence of blebbistatin, no SOCs are observed and the ESCRT-III subunit CHMP4B fails to polymerize from the midbody toward the presumptive abscission site. This likely explains the abscission delay and late ICB regression observed upon blebbistatin treatment. The authors conclude that the forces generated by myosin II define the future abscission sites (Wang et al., 2019).

Inhibition of myosin II activity also leads to abnormal accumulation of myosin IIA/B/C and F-actin at the ICB (Wang et al., 2019). Thus, myosin II-dependent contractility also favors F-actin disassembly from the ICB, possibly by sliding actin filaments which makes them more accessible to the actin clearance machinery (Wang et al., 2019), such as cofilin and the oxidoreductase MICAL1 (Lenhart and DiNardo, 2015; Fremont et al., 2017a; Fremont et al., 2017b; Bai et al., 2020; Niu et al., 2020; Iannantuono and Emery, 2021). This is likely important since F-actin accumulation prevents ESCRT-III polymerization (Dambournet et al., 2011; Schiel et al., 2012; Terry et al., 2018), perhaps because actin acts as a physical barrier that impairs interactions between the plasma membrane and ESCRT-III filaments, or favors a local high tension in the ICB detrimental for ESCRT-III assembly.

Taken together, the results presented above suggest that the actomyosin-dependent contractility plays several roles in the steps preceding abscission: first at the whole cellular level, by promoting cell migration and spreading which is believed to make the ICB thinner (Burton and Taylor, 1997; Bottcher et al., 2009; Wang et al., 2019) and second at the local ICB level, by generating SOCs and by contributing to F-actin clearance. Yet, the mechanism by which myosin II and F-actin sequentially appear and disappear from these sites is unknown. Of note, the SOCs likely correspond to “constriction sites” (CS) previously described by others, and reported to contain anillin and to contribute to ICB thinning (Renshaw et al., 2014). It remains to be investigated whether and how SOCs/SCs mature into presumptive abscission sites, also named “secondary ingressions” and initially observed by a different lab (Schiel et al., 2012). Actually, F-actin is also detected at these secondary ingression sites before abscission (Dema et al., 2018). However, myosin II-mediated constriction is likely not sufficient to generate the secondary ingression sites, since other factors such as FIP3-positive endosomes and associated cargoes have been involved (Schiel et al., 2012; Fremont and Echard, 2018). These endosomes might change the lipid composition of the plasma membrane at the future abscission site and favor the formation of the ESCRT-III cone (see also Section 3.3). Thus, the respective role of contractile machineries, anillin and trafficking in the local definition of the future abscission site remains to be clarified.

#### 4.1.3 Decrease of actomyosin II-dependent contractility releases ICB tension and promotes abscission

ICB tension decreases before abscission and this tension release is required for proper ESCRT-III polymerization from the midbody to the abscission site in epithelial HeLa cells (see Section 1 and Section 2). Both membrane and cortical tension depend on actomyosin contractility and reducing myosin II activation could explain the observed drop of ICB tension. Consistently, inhibiting myosin II activation by treating the cells with the ROCK inhibitor Y27632 is both sufficient to fully abolish ICB tension and to considerably accelerate abscission (Lafaurie-Janvore et al., 2013). It is important to point out that this should be confirmed by direct inhibition of Myosin II with specific drugs, since the inhibition of ROCK also leads to cofilin inhibition, which could result in reduced actin filament turnover and severing. Alternatively, stopping daughter cell migration or establishment of new cell-cell junctions between dividing cells in crowded environments, as epithelia, would compress the ICB and reduce the ICB tension without necessarily reducing myosin II activity (Lafaurie-Janvore et al., 2013). Finally, proteomic approaches unexpectedly revealed that the ICB and midbody contain numerous myosin motors and actin binding proteins that could possibly modulate the ICB tension (Capalbo et al., 2019; Peterman et al., 2019; Addi et al., 2020; Rai et al., 2021).

### 4.2 Role of caveolae in regulating ICB tension and ESCRT polymerization

**FIGURE 4:**
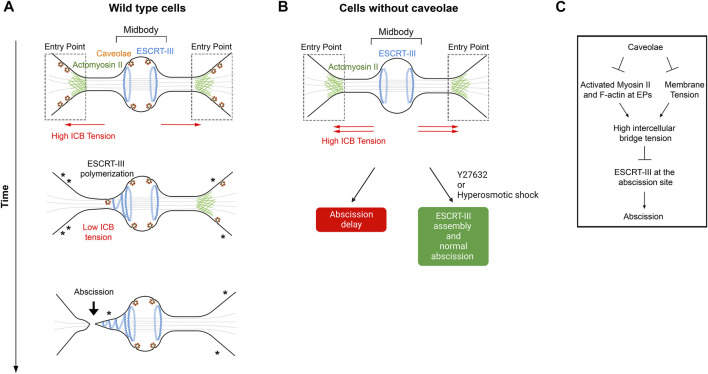
– Caveolae regulate ICB tension and ESCRT-III polymerization at the abscission site. **A**- Working model for caveolae-mediated regulation of the ICB tension. Note the progressive disappearance of caveolae (where asterisks are represented), possibly by flattening, at the Entry points while cells progress toward abscission. This membrane flattening is proposed to contribute to a decrease of the ICB tension, which promotes ESCRT-III polymerization and abscission. **B-** Depletion of caveolae leads to abscission delay, which can be corrected by either Y27632 (ROCK inhibitor) or hyperosmotic shock treatments. **C-** Schematic summary of events regulated by caveolae at the ICB. Adapted from (Andrade et al., 2022). EPs: Entry Points.

Caveolae are 50- to 100-nm invaginations of the plasma membrane (Palade, 1953; Yamada, 1955) and where recently reported to limit ICB tension and favor abscission in HeLa cells (Andrade et al., 2022). Caveolae are particularly present in cell types experiencing mechanical stress and play critical roles in intracellular signaling (Parton, 2018; Parton et al., 2020). In addition, through their ability to flatten and provide extra membrane, they play a pivotal role to buffer plasma membrane tension and to prevent membrane rupture in non-dividing cells that experience mechanical constraints (Gervásio et al., 2011; Sinha et al., 2011; Del Pozo et al., 2021). After furrow ingression, caveolae are found at the midbody, at the tip of the ESCRT-III cone where abscission presumably occurs, and, at high density, at the EPs defined in Section 2.1 as the interface between the ICB and the cell bodies (Andrade et al., 2022) (Figure 4A). Depletion of the key caveolae component Cavin1 results in a complete loss of caveolae and leads to increased binucleation and delayed abscission due to defective ESCRT-III polymerization at the midbody and at the abscission site. This is associated with the persistent accumulation of F-actin and myosin II specifically at the EPs, where caveolae are normally present. This suggests that caveolae, directly or indirectly, locally limit actomyosin contractility. Furthermore, the abnormally prominent actomyosin pools at EPs in caveolae-depleted cells are likely responsible for the observed increase in tension at the ICB and the abscission defects. Indeed, lowering the cell tension with small doses of the ROCK inhibitor Y27632 1) decreases the percentage of ICBs with activated myosin II at the entry points to control values; 2) restores normal tension at the ICB; 3) corrects the localization of ESCRT-III at the abscission site and 4) restores normal abscission (Figure 4B). Of note, in interphase cells, caveolae-depleted cells show increased membrane tension, as measured by the Flipper-TR probe (Roffay et al., 2021). Consistently, reducing membrane tension by a short hyperosmotic shock during cytokinesis is sufficient to restore normal ESCRT-III polymerization at the abscission site in caveolae-depleted cells, arguing that both membrane tension and cortical tension at the ICB are regulated by the presence of caveolae in wild type cells. Overall, this study suggests that caveolae limit the ICB tension in normal dividing cells by the following scenario: when daughter cells respread on the substrate, they pull on their ICB, which is thus under tension. The presence of actin and myosin II at EPs, which is observed right after furrow ingression and might result from tension-induced myosin II recruitment—as shown previously in *Dictyostelium* (Effler et al., 2006)—could further increase the ICB tension and favor ICB thinning. In response to the ICB tension, the caveolae strongly present at EPs progressively flatten to release the ICB tension, which favors ESCRT-III recruitment. By limiting the ICB tension, caveolae thus promote normal abscission (Figure 4C).

### 4.3 Lipid remodeling at the ICB can modify the mechanical properties of the ICB membrane during cytokinesis

Membrane tension is an important component of the measured ICB tension (Lafaurie-Janvore et al., 2013) and reflects how the membrane can resist to an increase in membrane area (Sens and Plastino, 2015). The force needed to deform a membrane, for instance by the ESCRT-III machinery, depends both on 1) the membrane tension and 2) the membrane bending rigidity or stiffness (reflected by its bending modulus Kb) which depends on the lipid and protein composition. Mass spectrometry of cytokinetic cells revealed an enrichment of specific lipid species at the ICB, as compared to interphase or mitotic cells, indicating that cells change their lipidome throughout cell division (Atilla-Gokcumen et al., 2014; Arai et al., 2015). Interestingly, supported lipid bilayers of lipid preparations from cytokinetic cells are stiffer than the ones from interphase cells, demonstrating differences in the mechanical membrane properties during cytokinesis. This was measured by the force needed to breakthrough the bilayer using AFM, which was 3 to 6-times higher for cytokinetic lipids than for the interphase ones (Atilla-Gokcumen et al., 2014). Enrichment of ceramides, glycosphingolipid GM1, cholesterol and phosphoinositides at the ICB suggests a specific function of these lipids during cytokinesis, and several lipid enzymes remodel these lipids, e.g., phosphoinositides, before abscission (Dambournet et al., 2011; Cauvin and Echard, 2015; Gulluni et al., 2021). Some of these lipid changes directly help the recruitment of ESCRT components—such as the ESCRT-II subunit VPS36 by PI(3,4)P2 (Gulluni et al., 2021)—but could also locally change the membrane stiffness. Although not demonstrated during cytokinesis, one can speculate that a reduction of the membrane bending modulus by modifying lipids at the ICB would favor membrane deformability, thus ESCRT-III assembly and polymerization, as reported for the drop in membrane tension. Actually, mechanical rigidity of the membrane is an important parameter to take into account in models that explain how ESCRT-III can reshape membranes (Harker-Kirschneck et al., 2019, Meadowcroft et al.). Of note, for a membrane of a given stiffness, the sequential copolymerization of different ESCRT-III subunits with increasing adhesion energies is predicted to be crucial for membrane deformation, as recently observed *in vitro* (Meadowcroft et al., Pfitzner et al., 2020; Pfitzner et al., 2021).

Similarly, a reduction of the ICB diameter at the future abscission site by means described in Section 3.1 or by specific changes in the shape of the membrane lipids could increase the negative local curvature (perpendicular to the long axis of the ICB) which generally favors ESCRT binding (Schoneberg et al., 2017; McCullough et al., 2018).

### 4.4 An emerging connection between ICB tension and the abscission checkpoint

The abscission checkpoint (also referred as the NoCut checkpoint) is an evolutionarily conserved pathway in eukaryotic cells that delays abscission in response to various signals including the abnormal presence of chromatin bridges in the ICB, replication stress and nuclear pore defects (reviewed recently in (Petsalaki and Zachos, 2021a; Hong et al., 2021)). The activation of the checkpoint (Steigemann et al., 2009; Mackay et al., 2010; Bhowmick et al., 2019) leads to the activation of the Aurora B kinase and its translocation to the central part of the midbody, which eventually both stabilizes the ICB through actin polymerization (Steigemann et al., 2009; Dandoulaki et al., 2018; Bai et al., 2020) and inhibits the polymerization of ESCRT-III filaments at the abscission site—in particular through CHMP4C phosphorylation and VPS4 sequestration (Capalbo et al., 2012; Carlton et al., 2012; Thoresen et al., 2014; Caballe et al., 2015; Capalbo et al., 2016) (Figure 3, right panel). In the case of chromatin bridges, it is believed that this gives extra time and opportunity for the connected daughter cells to resolve the DNA bridge and restore equal segregation of the genetic information. In the presence of this particular stress, checkpoint deficiency results in either binucleation and tetraploidy, or chromatin bridge breakage, both leading to DNA damage and chromosome instability associated with increased cancer susceptibility (Carlton et al., 2012; Amaral et al., 2016; Hong et al., 2018; Sadler et al., 2018; Lens and Medema, 2019). Full activation of Aurora B at the midbody upon checkpoint activation depends on several inputs, some of which likely sense the presence of chromatin (Mackay and Ullman, 2015; Petsalaki and Zachos, 2016; Fung et al., 2017; Petsalaki and Zachos, 2021b). While most studies focused on the activation and identification of activators of the checkpoint activated by chromatin bridges, two studies suggest that ICB tension induces this signaling pathway to delay cytokinetic abscission.

In a first study, the kinase ULK3 (Unc-51 like kinase 3) was shown to bind and phosphorylate several ESCRT-III components: IST1, CHMP1A/B and CHMP2A (Caballe et al., 2015). This kinase is a component of the abscission checkpoint since the delay observed upon checkpoint activation by nuclear pore defects and chromatin bridges depends on the presence of ULK3 in HeLa cells. By phosphorylating IST1, ULK3 induces the relocation of CHMP4B and IST1 to the central zone of the midbody and increases VPS4 interaction with IST1, thus explaining the delayed abscission. In addition, ULK3 activation depends on both Aurora B kinase activity and on CHMP4C. Interestingly, in the absence of chromatin bridges, the delay in abscission observed when cells are cultured at low density is also dependent on the presence of ULK3. All together, these results suggest that high ICB tension found in cells cultured at low density activates the abscission checkpoint (or at least some of the components, namely ULK3) to delay abscission (Caballe et al., 2015). Noteworthy, the effector of ULK3 in response to high tension is not IST1 and remains to be identified (Caballe et al., 2015). It could potentially be CHMP4C, CHMP1A/B, CHMP2A or another unknown protein (Figure 3)**.**

In a recent report, the authors observed the striking apparition of cytosolic “Abscission Checkpoint Bodies” (ACBs) in response to the abscission checkpoint activation by nuclear pore defects and replication stress—but not by chromatin bridges—in cytokinetic HeLa cells and RPE1 cells (Williams et al., 2021). In cells with nuclear pore defects, these ACBs contain several proteins involved in abscission: CHMP4B, ALIX, activated Aurora B and pppCHMP4C (Capalbo et al., 2016; Williams et al., 2021). Concomitantly, a delay in ALIX, TSG101 and IST1 recruitment at the midbody is observed, which likely contribute to the delayed abscission. Interestingly, a two-fold reduction of ALIX levels in early ICBs were also observed in cytokinetic cells cultured at low density (high tension), compared to cells cultured at high density (low tension) (Williams et al., 2021). Furthermore, a small but significant increase of the proportion of cells with activated Aurora B-positive ACBs was measured in cytokinetic cells at low density, compared to high density (Williams et al., 2021). Thus, ACBs appear both in cytokinetic cells with nuclear pore defects and in cells with high ICB tension (Williams et al., 2021).

To summarize, there is emerging evidence that high ICB tension associated with low density culture conditions activate the abscission checkpoint. However, recent observations argue that it may not always be the case. Indeed, caveolae-depleted cells also display high tension (see Section 3.2) and abscission defects that can be rescued by culturing these cells at high density, yet, there is no apparent sign of activation of the abscission checkpoint (Andrade et al., 2022). The origin of the high ICB tension (membrane tension vs. cortical tension or MT pulling) might differentially activate the checkpoint, but this remains to be investigated.

## 5 Discussion

We discuss below some of the questions which, in our opinion, would be important to answer to better understand the role of mechanical inputs in abscission.

### 5.1 Tension-regulated ESCRT-III regulation of abscission: is this always the case?

Most of what we have learnt so far regarding the role of mechanics in the regulation of cytokinetic abscission is based on isolated cultured, epithelial HeLa cells. There are several lines of evidence showing that the rules observed in these cells—such as inhibition of ESCRT-III assembly when the ICB is under tension—do not necessarily apply to all cells (such as highly migrating cells and/or non cancer cells) or in particular mutant contexts. Indeed, recent work raises the possibility that ESCRT-III independent abscission mechanisms exist in mammalian cells, based on the study of Cep55 KO mice (Tedeschi et al., 2020; Little et al., 2021). The recruitment of the ESCRT machinery is absent in a high fraction of ICBs in Cep55 KO cells and, unexpectedly, it was found that Cep55 is not essential for cell division in most tissues. Of note, ALIX, TSG101 and ESCRT-III components are not detected in most (66%) wild type fibroblasts, and fibroblasts depleted of ESCRT-III components can divide (Tedeschi et al., 2020). As initially proposed (Burton and Taylor, 1997), these data suggest a possible mechanical rupture of the ICB when cells migrate apart over sufficiently long distances (see the presented movies in (Tedeschi et al., 2020)). Whether this is the mechanism of abscission for these cells and other cell types *in vivo*, in wild type or in Cep55 KO mice, remains to be investigated. Interestingly, depending on the stiffness of the environment, it is possible that migration-driven, rather than ESCRT-driven abscission could be favored (Gupta et al., 2018; Rabie et al., 2021). This concept of “traction-mediated cytoplasmic fission” or “cytofission” based on crawling on a stiff substratum was actually proposed a long time ago, as it is one way *Dictyostelium discoideum* uses to physically divide when the gene encoding myosin II is genetically disrupted (De Lozanne and Spudich, 1987; Neujahr et al., 1997). Of note, this “illegitimate cell division” (De Lozanne and Spudich, 1987) is independent of mitosis, does not rely on the formation of a midbody but requires F-actin (Neujahr et al., 1997) and depends on Arp2/3-dependent migration (King et al., 2010). Remarkably, migration-dependent cytofission can also be observed in human HT1080 (fibroblasts) and RPE1 (retinal pigment epithelial cells) cells when the actomyosin ring function is compromised (Kanada et al., 2008; Dix et al., 2018). RPE1 cells without functional contractile ring must adhere to a stiff-enough substrate *via* β1-integrins and migrate apart after chromosomal segregation to physically split into two independent daughter cells (Dix et al., 2018). Importantly, cytofission seems to be also relevant in mouse 3T3, cells, human RPE1, B16 melanoma and MCF10 A cells in the absence of actomyosin ring defects but after a previously failed cytokinesis (Ben-Ze’ev and Raz, 1981; Choudhary et al., 2013). Indeed, binucleated cells seeded on a substrate split themselves into 2 cells through opposite migration of the cytoplasm of the mother cell during G1, leading to the correct number of chromosomes in the progeny. Again, no midbody markers (PLK1 and MKLP1) are found in the cytoplasmic bridges and this process is dependent on myosin II and actin (Choudhary et al., 2013). Thus, cytofission is likely an ESCRT-III independent process and could represent an ancient and evolutionarily conserved mechanism of abscission particularly important to preserve genomic integrity in “emergency situations”. There is thus an urgent need to study the mechanisms of abscission in a wider range of unperturbed cells and to identify whether tension-dependent inhibition of ESCRT-III recruitment at the midbody applies only to a subset of epithelial cells or beyond.

### 5.2 Does global vs. local actomyosin contractility determine the ICB tension?

We discussed several actomyosin pools that could control the ICB tension: the cell body cortical pool, the pool at the constriction sites and the pool at the EPs close to the ICB. To address the function of these different pools, new optogenetic tools could be used to locally inhibit myosin II (Yamamoto et al., 2021). If the actomyosin pool at the EP is key for regulating the ICB tension (Andrade et al., 2022), its inhibition should trigger ESCRT-III polymerization at the abscission site, as observed upon laser ablation. It was noticed that one pool of caveolae was systematically higher in one of the two daughter cells and correlates with the side of the first abscission (Andrade et al., 2022). The asymmetric inactivation of myosin II in only one of the two EPs might thus control on which side of the midbody abscission first occurs.

### 5.3 How caveolae regulate the ICB tension during cytokinesis?

Caveolae are proposed to regulate the ICB tension by two non-mutually exclusive and perhaps intimately-linked mechanisms (Andrade et al., 2022) (Figure 4C). First, by flattening out at the EPs and at the tip of the ESCRT-III cone caveolae could contribute to reduce membrane tension at the ICB, as previously demonstrated and quantitatively modelized in non-dividing cells (Sens and Turner, 2006; Sens and Plastino, 2015). Second, caveolae could reduce actomyosin activity at the EPs since their depletion leads to a local increase of activated myosin II and F-actin accumulation, through a mechanism the remains to be discovered (Andrade et al., 2022). Interestingly, increasing evidence suggest a tight relationship between caveolae and the actin cytoskeleton, as well as a role of caveolae in controlling both membrane tension and local cortical contractility (Grande-García et al., 2007; Goetz et al., 2011; Echarri and Del Pozo, 2015; Echarri et al., 2019; Hetmanski et al., 2019; Domingues et al., 2020; Teo et al., 2020). This has been recently studied in melanin transfer from melanocytes to keratinocytes (Domingues et al., 2020) and in rear to front retraction during durotactic migration (Hetmanski et al., 2019). Furthermore, caveolae regulate epithelial monolayer tension to successfully extrude oncogenic cells (Teo et al., 2020), through possibly very similar mechanisms at stake during cytokinesis. Indeed, loss of caveolae upon caveolin-1 depletion leads to enhanced tension at adherens junctions in epithelia, as shown by increased recoiling speed after laser ablation (Teo et al., 2020). In addition, these defects can be rescued by ROCK inhibition and are associated with a local increase of F-actin levels. Mechanistically, caveolae depletion was proposed to release free the phosphoinositide PtdIns(4,5)P2 to promote actin polymerization locally through FMNL2 formin recruitment at the plasma membrane (Teo et al., 2020). Whether this is also the case at EPs in caveolae-depleted cells during cytokinesis has to be explored. Altogether, this suggests that caveolae could locally inhibit actomyosin contractility and thereby regulate tension both in dividing and non-dividing cells.

### 5.4 How is the ICB tension sensed? Is the abscission checkpoint involved?

A simple way to sense the ICB tension is to use mechanosensitive molecular complexes, such as caveolae. Caveolae at the EPs could be ideally positioned to sense the ICB tension, since the ICB/cell interface is likely under high tension, as indicated by its funnel shape (Andrade et al., 2022) (Figure 4A). The tension could also be directly sensed by the ESCRT machinery, with lower membrane tension and higher negative curvature favoring its assembly at the ICB membrane.

Alternatively, the ICB tension could be sensed by the abscission checkpoint and trigger a biochemical signaling cascade that eventually inhibits the ESCRT machinery (Figure 3, right panels). Aurora B might be activated by high tension between overlapping MTs at the midbody if the ICB is itself under mechanical tension. To test the connection between the checkpoint and ICB tension, it would be important to experimentally address the following questions: does active Aurora B translocate to the central part of the MB upon high ICB tension (e.g., low cell density conditions)? Which ULK3 effectors inhibit the polymerization of the ESCRT machinery under high ICB tension? Are the kinases ATM, Chk2, Clk1/2/4, known to activate and localize Aurora B upon checkpoint activation by nuclear pore defects or chromatin bridges (Petsalaki and Zachos, 2016; Petsalaki and Zachos, 2021b) activated by high ICB tension? Are the proteins known to regulate actin polymerization at or close to the ICB in the context of the abscission checkpoint (e.g., Src and MsrB2 (Dandoulaki et al., 2018; Bai et al., 2020)) also involved under high ICB tension? Finally, why do different situations with high ICB tension activate (low density conditions) or not (caveolae depletion) the abscission checkpoint (Caballe et al., 2015; Williams et al., 2021; Andrade et al., 2022)?

### 5.5 Mechanical regulation of abscission: can it be measured in vivo, within tissues? What could be its physiological relevance?

To our knowledge, there is no study that has measured tension in the ICB in the context of a tissue, *in situ*. The influence of neighboring cells on furrow ingression and midbody positioning *in vivo* or in *vitro* tissues has started to be investigated (e.g., (Campinho et al., 2013; Founounou et al., 2013; Guillot and Lecuit, 2013; Herszterg et al., 2013; Mathieu et al., 2013; Morais-de-Sá and Sunkel, 2013; Wyatt et al., 2015; Cao et al., 2017; Pinheiro et al., 2017; Daniel et al., 2018; Uroz et al., 2019; Adar-Levor et al., 2021; Little et al., 2021; Mathieu et al., 2022)). However, measuring mechanical parameters *in vivo* is technically challenging. The use of chemical probes for membrane tension such as Flipper-TR might be helpful (Colom et al., 2018) but caution should be exercised since both membrane tension and lipid membrane composition influence the fluorescence.

Functionally, the inhibition of ESCRT-III assembly by ICB tension could ensure that 1) the abscission machinery is recruited only after pulling forces have promoted thinning of the ICB, and 2) abscission occurs only when cell-cell junctions between daughter cells have been re-established—a phenomena that likely releases ICB tension—in order to preserve epithelial tissue integrity (Lafaurie-Janvore et al., 2013). Finding mutant situations that reduce the ICB tension *in vivo* could demonstrate whether tissue permeability and organization is disrupted and help understand the physiological relevance of triggering abscission only after a release of the ICB tension.

Answering these questions will require the development of new tools to measure and perturb tension *in vivo* and call for studies in more diverse cell types and models. This should help understand the role of mechanics in cytokinetic abscission, in particular in a multicellular context, and the advantage of a mechanically-regulated abscission compared to a simple mechanical rupture.
